# Transcription factors in microcephaly

**DOI:** 10.3389/fnins.2023.1302033

**Published:** 2023-11-29

**Authors:** Youngshin Lim

**Affiliations:** ^1^Department of Pathology and Laboratory Medicine, Cedars-Sinai Medical Center, Los Angeles, CA, United States; ^2^Department of Biomedical Science Education, Charles R. Drew University of Medicine and Science, Los Angeles, CA, United States

**Keywords:** brain size, cortical development, microcephaly, proliferation, neurogenesis, transcription factors

## Abstract

Higher cognition in humans, compared to other primates, is often attributed to an increased brain size, especially forebrain cortical surface area. Brain size is determined through highly orchestrated developmental processes, including neural stem cell proliferation, differentiation, migration, lamination, arborization, and apoptosis. Disruption in these processes often results in either a small (microcephaly) or large (megalencephaly) brain. One of the key mechanisms controlling these developmental processes is the spatial and temporal transcriptional regulation of critical genes. In humans, microcephaly is defined as a condition with a significantly smaller head circumference compared to the average head size of a given age and sex group. A growing number of genes are identified as associated with microcephaly, and among them are those involved in transcriptional regulation. In this review, a subset of genes encoding transcription factors (e.g., homeobox-, basic helix-loop-helix-, forkhead box-, high mobility group box-, and zinc finger domain-containing transcription factors), whose functions are important for cortical development and implicated in microcephaly, are discussed.

## 1 Introduction

Microcephaly is a clinical condition where the occipito-frontal head circumference (OCF) of an individual is significantly smaller than the average head size of the population for a given sex and age (Lim and Golden, 2020). Typically, a cutoff of more than 2 standard deviations (SDs) below the mean or less than the 3rd percentile is used for microcephaly diagnosis; however, other cutoff values such as more than 3 SD and less than the 5th or 10th percentile can be used as well (Opitz and Holt, 1990; Raymond and Holmes, 1994; Ashwal et al., 2009). The prevalence of microcephaly is between 1.5 and 8.7 per 10,000 births in Europe and the US, respectively (Cragan et al., 2016; Morris et al., 2016), and ~15–20% of children with developmental delay are reported to have microcephaly (Sassaman and Zartler, 1982; Watemberg et al., 2002; Aggarwal et al., 2013). Microcephaly can manifest as the only phenotype without other obvious morphologic or functional abnormalities (e.g., cortical- or extra-cortical malformations, and intellectual disability), as in the case of “isolated microcephaly”; or it can be accompanied by neurological or psychiatric conditions, but without cortical or extra-cortical malformations, as in the case of “non-syndromic microcephaly”. In contrast, “syndromic microcephaly” often presents together with other cortical malformations (e.g., polymicrogyria, lissencephaly, periventricular nodular heterotopia, and agenesis of corpus callosum) or can be part of a broader syndrome involving other organ systems (more than 700 genetic syndromes are reported to have microcephaly) (Pirozzi et al., 2018).

Depending on the time of presentation, microcephaly can be classified as primary (congenital) or secondary (postnatal) microcephaly. Congenital microcephaly is usually caused by deficiencies in the number of neurons (either reduced production or increased loss), while postnatal microcephaly is more often attributed to defective brain growth and/or neurodegeneration causing brain atrophy (Gilmore and Walsh, 2013). However, this classification is by no means strict. For example, a defect in neuronal generation can still result in postnatal microcephaly if the proliferation defect is not severe enough to manifest at birth.

The recent explosion in the use of next-generation sequencing has enabled the discovery of many novel disease-causing genetic variants in microcephaly. For example, 32 primary microcephaly (MCPH, microcephaly primary hereditary) genes have been identified (Asif et al., 2023), and the list is rapidly growing. Most of these genes encode centrosome-specific proteins, spindle-associated proteins, microtubule-associated proteins, and cell cycle checkpoint proteins (e.g., *ASPM, CDK6, CDK5RAP2, CENPJ, CEP63/135/152, NIN, PLK4, STIL, TUBGCP6*, and *WDR62*, among others) that cause cell cycle-related defects when mutated (Lim and Golden, 2020). Another group of the prominent MCPH genes encode proteins in various DNA damage responses and repair pathways (e.g., *PNKP, ERCC6, ERCC8, CHEK2, NHEJ1, XRCC4, XRCC5*, and *XRCC6*, among others) (Lim and Golden, 2020). In addition, the list also includes genes encoding proteins involved in transcriptional regulation (e.g., transcription factors and chromatin remodeling proteins), although only a small number of them are identified in MCPH (Jayaraman et al., 2018). If syndromic microcephalies are also considered, many additional transcription-related proteins associated with microcephaly can be included (see below).

In this review, transcription factors associated with microcephaly will be discussed, focusing on their reported functions and pathogenic mechanisms in model systems, which can be linked to clinical features in humans. Selected members of five different transcription factor families will be highlighted, including (1) homeobox genes such as *ARX, LHX2, MEIS, NKX2-1, OTX1, OTX2*, and *PAX6*; (2) bHLH (basic helix-loop-helix) genes such as *MYCN* and *TCF4*; (3) FOX (forkhead box) genes, *FOXG1* and *FOXR1*; (4) SOX (sex-determining regions Y-related HMG box) genes belonging to *SOXB1* (*SOX2* and *SOX3*) and *SOXC* (*SOX4* and *SOX11*) subfamily; and finally (5) zinc finger genes, *ZNF238* and *ZNF355* (Table 1). While not covered in this review, chromatin remodeling proteins are also critical factors in transcriptional regulation that can result in microcephaly when mutated. For instance, the *SMARCB1, SMARCA4, SMARCE1, ARID1A*, and *ARID1B* genes encode subunits of the BAF complex (also known as the SWI/SNF complex in yeast), and they are responsible for 55–70% of Coffin–Siris syndrome cases, where microcephaly can manifest as one of the phenotypes (Kosho et al., 2014; Vergano et al., 2021).

**Table 1:** Transcription factors associated with microcephaly.

**Gene family**	**Gene name**	**Chromosomal location**	**Microcephaly**	**Other major clinical conditions**	**Associated disease/syndrome**
Homeobox	***ARX***	Xp22.13	Congenital and postnatal (Kato et al., 2004; Kwong et al., 2019)	Lissencephaly, agenesis of corpus callosum, epilepsy, intellectual disability, autism spectrum disorder symptoms	XLAG, HYD-AG, Proud syndrome, Ohtahara syndrome, IEDE, XMESID, Partington syndrome, etc.
	***LHX2***	9q33.3	Postnatal (Schmid et al., 2023)	Intellectual disability, autism spectrum disorder, dysgenesis of corpus callosum, ophthalmologic abnormalities	
	***MEIS2***	15q14	Congenital and postnatal (Verheije et al., 2019)	Palatal and heart defects, dysmorphic facial features, intellectual disabilities	
	***NKX2.1***	14q13	Postnatal (Carré et al., 2009)	Benign hereditary chorea, respiratory symptoms, congenital hypothyroidism	Brain–lung–thyroid syndrome
	***OTX1***	2p15	Congenital (Liang et al., 2009)	Growth retardation, congenital hypopituitarism, optic atrophy	
	***OTX2***	14q22.3	Congenital (Gregory et al., 2021)	Congenital hypopituitarism, eye defects	
	***PAX6***	11p13	Congenital (Glaser et al., 1994; Schmidt-Sidor et al., 2009; Solomon et al., 2009)	Severe brain malformation, intellectual disability, autism, impaired audition, and eye defects	
bHLH	***MYCN***	2p24.3	Congenital (Courtens et al., 1997; Bokhoven et al., 2005; Marcelis et al., 2008)	Gastrointestinal atresia, learning disability, facial dysmorphism, syndactyly, and cardiac defect	Feingold syndrome
	***TCF4***	18q21.2	Postnatal (Zweier et al., 2008; Winter et al., 2016; Goodspeed et al., 2017)	Developmental delays, intellectual disability, characteristic facial features, speech delay, sleep disturbance, autism spectrum disorder symptoms, seizures, and severe myopia.	Pitt–Hopkins syndrome
Forkhead box	***FOXG1***	14q12	Congenital (Hou et al., 2020)	Agenesis of the corpus callosum, delayed myelination, intellectual disability, autism spectrum disorder symptoms, epilepsy	FOXG1 syndrome (used to be considered as a congenital variant of Rett syndrome)
	***FOXR1***	11q23.3	Postnatal (Mota et al., 2021)	Progressive brain atrophy, global developmental delay, variety of carcinomas	
SRY-related HMG box	***SOX2***	3q26.33	Postnatal (Fantes et al., 2003; Schneider et al., 2009; Blackburn et al., 2018)	Severe eye defects, hippocampal abnormalities, epilepsy, motor problems, dysmorphic facial features, and genital anomalies	
	***SOX3***	Xq27.1	Postnatal (Jelsig et al., 2018; Li et al., 2022)	Hypopituitarism (from isolated growth hormone deficiency to panhypopituitarism), intellectual disability, neural tube defects, and craniofacial abnormalities	
	***SOX4***	6p22.3	Postnatal (Zawerton et al., 2019; Angelozzi et al., 2022)	Intellectual disability, facial dysmorphism, 5th finger and/or toe anomalies	Coffin–Siris syndrome
	***SOX11***	2p25.2	Congenital (Tsurusaki et al., 2014; Hempel et al., 2016; Wakim et al., 2020)	Growth deficiency, intellectual disability, characteristic facial features, and hypoplastic nails of the fifth fingers and/or toes	Coffin–Siris syndrome
Zinc finger	**ZNF238** ^*^	1q44	Congenital and postnatal (Boland et al., 2007; Hill et al., 2007; Bon et al., 2008; Orellana et al., 2010; Khadija et al., 2022)	Severe intellectual disability, profound growth defects, short stature, agenesis of corpus callosum, dysmorphic facial features	1qter deletion syndrome
	***ZNF335***	20q13	Congenital (Yang et al., 2012)	Simplified gyrification, thinned cerebral cortex, abnormal lamination, death by 1 year of age	

* ZNF238 is one of the several genes deleted in 1qter deletion syndrome.

XLAG, X-linked lissencephaly with abnormal genitalia; HYD-AG, hydranencephaly and abnormal genitalia; IEDE, idiopathic infantile epileptic-dyskinetic encephalopathy; XMESID, X-linked myoclonic epilepsy with spasticity and intellectual disability.

## 2 Transcription factors associated with microcephaly

### 2.1 Homeobox genes: *ARX, LHX2, Meis, NKX2-1, OTX1, OTX2*, and *PAX6*

**Figure 1:**
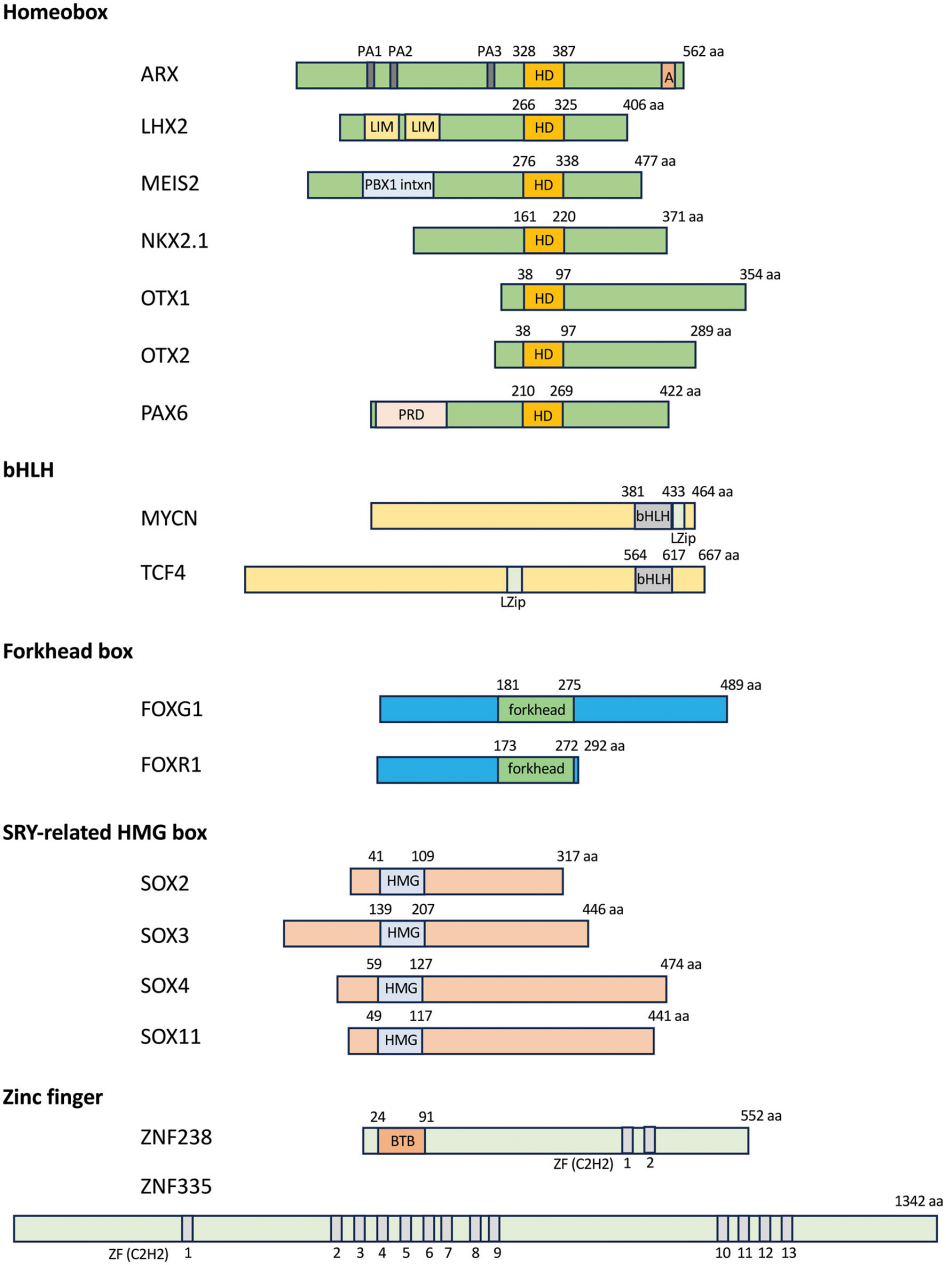
Transcription factors associated with microcephaly. Schematics of the domain structure of the homeobox, bHLH, forkhead box, HMG box, and zinc finger transcription factors. Numbers over the DNA-binding domain of each selected transcription factor indicate amino acid residues, while numbers below the gray domains in the zinc finger genes indicate each C2H2 zinc finger domain. A, aristaless domain; BTB, BTB domain [Broad-Complex, Tram track, and Bric-a-brac, also known as POZ (poxvirus zinc finger) domain; bHLH, basic helix-loop-helix domain; C2H2, cysteine–cysteine–histidine–histidine; forkhead, forkhead box domain; HD, homeobox domain; HMG, high mobility group; LIM, LIM domain (Lin-11, Isl1, and Mec-3); LZip, leucine zipper domain; PA1-3, poly alanine track 1–3; PBX1 intxn, PBX1 interaction domain; PRD, paired domain; ZF, zinc finger domain.

The homeobox gene family members are characterized by 180-bp DNA sequences known as the “homeobox” encoding a 60-amino acid protein domain called the “homeodomain” (Figure 1). Most homeodomains contain a helix-loop-helix-turn-helix structure responsible for DNA binding (Gehring et al., 1994; Bürglin and Affolter, 2016). The DNA-binding motif is typically located at the second or third helix and recognizes and binds to the major groove of DNA at specific consensus sites (Kappen, 2000). The proteins encoded by homeobox genes are called homeodomain proteins, or simply homeoproteins. In addition to the homeodomain, homeoproteins often contain other domains or motifs (e.g., paired domain and “PRD”) that can contribute to DNA and/or co-factor binding (Leung et al., 2022). Often, these additional DNA-binding domains and co-factor binding sites confer additional DNA specificity to these proteins, apart from the consensus binding sequence (Holland et al., 2007). Homeoproteins can be divided into various different classes, such as the Antennapedia (ANTP), Paired (PRD), and LIM classes (Holland et al., 2007), based on the variations in the homeodomain and additional domains or motifs. Well-known examples of homeoproteins belonging to these three classes include HOX (ANTP class), PAX (PRD class), and LHX (LIM class) (Holland et al., 2007).

**Figure 2:**
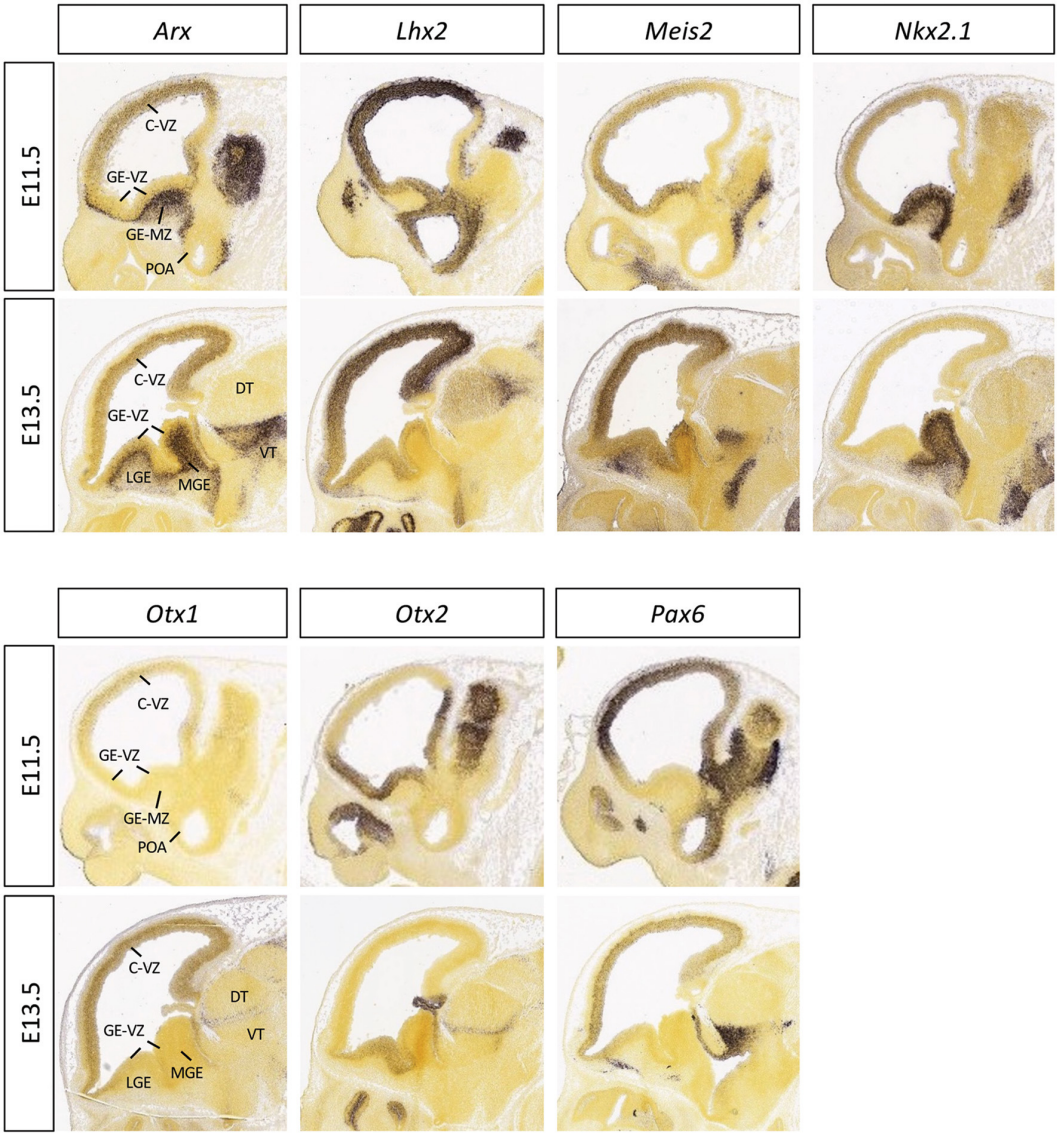
Expression patterns of the selected homeobox transcription factors in embryonic mouse brain. The mid-sagittal sections of E11.5 or E13.5 embryonic brains showing RNA *in situ* hybridization results of *Arx, Lhx2, Meis2, Nkx2.1, Otx1, Otx2*, and *Pax6*, adapted from Allen Brain Atlas (https://developingmouse.brain-map.org).

Mutations in homeobox genes were first described in *Drosophila*, which resulted in homeotic transformation (normal structure developing at an abnormal body position, e.g., leg growing from the location expected to be antennae) (Gehring et al., 1994). It is now known that homeoproteins control many cellular processes crucial for embryonic development, including proliferation, differentiation, apoptosis, cell shape, cell adhesion, and migration (Pearson et al., 2005). Furthermore, homeobox genes are known to be associated with many developmental brain disorders and cancers in humans (Leung et al., 2022). In this study, I focus on several homeobox genes (*ARX, LHX2, MEIS2, NKX2-1, OTX1, OTX2*, and *PAX6*) implicated in the pathogenesis of microcephaly (Table 1). The expression patterns of these homeobox genes in the embryonic mouse brains (Figure 2), their functional roles during brain development, and their human genetic variants and clinical features associated with microcephaly will be highlighted.

#### 2.1.1 *ARX* (Aristaless-related homeobox)

Aristaless-related homeobox gene (*ARX*) belongs to the PRD class, one of the three largest classes of homeobox genes. It is the vertebrate homolog of *Drosophila aristaless* (*al*), essential for the formation of the head segment and the axis specification of appendages (Miura et al., 1997). It is located on the X chromosome (Xp22.13 in humans), and mutations in *ARX* are associated with a spectrum of neurodevelopmental conditions that can be classified into two groups—those with and those without structural abnormalities. The malformation group can include lissencephaly, microcephaly, and/or agenesis of corpus callosum and is associated with several syndromes: X-linked lissencephaly with abnormal genitalia (XLAG), hydranencephaly and abnormal genitalia (HYD-AG), and Proud syndrome (agenesis of corpus callosum with abnormal genitalia) (Dobyns et al., 1999; Ogata et al., 2000; Kitamura et al., 2002; Kato et al., 2004). The second group of *ARX*-associated conditions, typically without severe malformations, frequently includes a variety of infantile and childhood seizure phenotypes and non-syndromic X-linked intellectual disability (Bienvenu et al., 2002; Strømme et al., 2002; Kato et al., 2003). In rare cases, these relatively less severe conditions can also present with brain malformations (e.g., microcephaly in a case with Ohtahara syndrome) (Absoud et al., 2010). More severe phenotypes harboring brain malformations seem to arise from premature termination mutations (large deletions, frameshifts, and nonsense mutations) or missense mutations in the homeodomain or nuclear localization sequences, while missense mutations outside of the homeodomain or mutations in polyalanine tracts (expansion or deletion) are associated with less severe phenotypes (Friocourt and Parnavelas, 2010).

Interestingly, individuals with *ARX* variants can have either congenital or postnatal microcephaly (Friocourt and Parnavelas, 2010). For example, XLAG patients with nonsense mutation (c.1117C<T (Q373X)) or with missense mutation (c.1561G<A (A521T)) in the aristaless domain tend to have congenital microcephaly (Kato et al., 2004), while XLAG patients with missense mutation (c.989G > A; p.Arg330) located in the nuclear localization sequence (NLS2) or with a deletion mutation (790delC) predicted to cause premature truncation were reported to have postnatal (progressive) microcephaly (Kwong et al., 2019). Of note, in some cases of postnatal microcephaly, the underlying pathogenic mechanism is distinct from that of the congenital microcephaly—proliferation defects in congenital vs. growth defects or neurodegeneration in postnatal microcephaly (Absoud et al., 2010). However, in other cases, it is possible that the embryonic proliferation defects, which are not severe enough to be detected at birth, can manifest as postnatal microcephaly.

The function of ARX in the brain is well reflected by its expression pattern. In the dorsal telencephalon (developing cortex), *Arx* is expressed in the proliferating cells located in the ventricular zone (VZ) and subventricular zone (SVZ) (Figure 2). However, its expression is turned off once cells exit the cell cycle and begin to migrate out of the VZ and SVZ (Lim et al., 2022). In contrast, in the ventral forebrain, *Arx* is not expressed in the VZ; instead, it is strongly expressed in the SVZ and mantle zone of the lateral ganglionic eminence (LGE) and medial ganglionic eminence (MGE) (Figure 2) and remains expressed in non-radially migrating neurons emanating from the MGE to the cerebral cortex (Lim et al., 2022). Interestingly, when *Arx* is genetically ablated in mice from the dorsal telencephalon, neural progenitor expansion is disrupted, mainly due to premature cell cycle exit (Colasante et al., 2015). The brains of these mice are smaller, resembling the microcephaly observed in humans with an *ARX* mutation; however, these mice do not develop seizures (Friocourt and Parnavelas, 2010; Colasante et al., 2015). In contrast, when *Arx* is abrogated from the ventral telencephalon, the brains are of normal size, but all of the male mice exhibit a range of seizures (Marsh et al., 2009).

What is the pathogenic mechanism for ARX mutations to result in microcephaly? It appears that ARX regulates the expansion of cortical progenitors by regulating the level of *Cdkn1c* expression (Colasante et al., 2015). CDKN1C (also known as p57/KIP2) regulates the G1-S transition of cortical progenitors (Sherr and Roberts, 1999). When overexpressed in mice, it promotes progenitor cell cycle exit and a transition from proliferation to differentiation, while its loss leads to increased proliferation and macrocephaly (Mairet-Coello et al., 2012). Targeted ablation of *Arx* in cortical progenitors leads to an abnormal increase in *Cdkn1c* expression (ARX normally represses *Cdkn1c* expression), resulting in premature cell cycle exit and depleting progenitor pools, leading to a reduced brain size. Furthermore, the loss of *Arx* disrupts PAX6 and TBR2 expression in progenitor cells (Lim et al., 2019). PAX6 and TBR2 are also transcription factors expressed in progenitor cells, where they also play a role in proliferation (PAX6 for radial glial progenitors; TBR2 for intermediate progenitors). Their decreased expression in *Arx* mutant mice appears to be a second contributing mechanism leading to the microcephaly (Lim et al., 2019).

#### 2.1.2 *LHX2* (LIM homeobox 2)

*LHX2* is a member of the LIM (Lin-11, Isl1, and Mec-3) class of homeobox gene family. The protein contains a homeodomain (for DNA binding) and two cysteine-rich LIM zinc finger domains (required for zinc binding and functioning as a modular protein-binding interface to mediate protein–protein interactions) (Feuerstein et al., 1994; Schmeichel and Beckerle, 1994). LHX2 can form multimeric complexes with other co-factors such as LIM-domain-binding-1 (LDB1) via the LIM domains, allowing homeodomain-mediated DNA binding to activate its target genes (Schmeichel and Beckerle, 1994; Agulnick et al., 1996; Breen et al., 1998; Kadrmas and Beckerle, 2004).

*LHX2/Lhx2* is a vertebrate ortholog of the *Drosophila* “selector” gene *apterous* (*Ap*) which is essential for wing development (Cohen et al., 1992). Selector genes are known to function cell-autonomously to specify cell identity while suppressing alternative fates (Lawrence and Struhl, 1996; Irvine and Rauskolb, 2001). *Lhx2*, which is expressed in cortical precursor cells but not adjacent cells (choroid plexus epithelium and cortical hem), has been shown to act as a selector gene in the developing mouse cerebral cortex, specifying cortical identity in a cell-autonomous fashion while suppressing hippocampal organizer fate (Mangale et al., 2008). *Lhx2* homozygous knockout mice are embryonic lethal, likely due to severe anemia. They also show forebrain hypoplasia along with defects in eye development (anophthalmia) (Porter et al., 1997). Furthermore, dorsal forebrain conditional *Lhx2* mutant mice have dramatically smaller cerebral cortices when compared to controls (Chou et al., 2009), again resembling with human microcephaly phenotype observed in patients with *LHX2* variants (Schmid et al., 2023).

Until recently, *LHX2* defects had not been linked to neurodevelopmental disorders, with the exception of a few variants described in a large study on developmental disorders (Kaplanis et al., 2020) and in an autism cohort (Zhou et al., 2022). However, *LHX2* haploinsufficiency has now been linked to variable neurodevelopmental disorders (Schmid et al., 2023); the authors identified *de novo* deletions (likely gene-disrupting) and missense variants in *LHX2* from 19 individuals (18 families) presenting with a variable neurodevelopmental phenotype, including microcephaly, intellectual disability, autism spectrum disorder, and other behavioral anomalies. Among 10 patients carrying likely pathogenic variants with occipito-frontal head circumference (OFC) records available, 7 patients showed microcephaly at the time of investigation (age ranging from 2 to 8 years; OFC at birth not available); 4 of them had deletions or nonsense variants (likely gene-disrupting), and 3 had missense variants in the LIM domain or homeodomain (Schmid et al., 2023). These data implicate *LHX2* as a causative gene for microcephaly. Functional analysis of these variants revealed altered subcellular localization (nucleolar accumulation) for two missense variants located in the homeodomain, impaired interaction with co-factor LDB1 for another variant located in the LIM domain, and impaired transcriptional activation for four missense variants. These data suggest a loss-of-function effect of *LHX2*. Importantly, the identification of more genetic cases in future will strengthen the argument for the role of *LHX2* in microcephaly.

#### 2.1.3 *MEIS2* (Myeloid ecotropic viral integration site 2)

*MEIS2* belongs to the three-amino acid-loop extension (TALE) superfamily of the HOX class of homeobox genes, a homolog of the *Drosophila homothorax* gene (Leung et al., 2022). MEIS2 appears to function as a HOX co-factor, which binds to HOX proteins or pre-B cell leukemia homeobox (PBX) transcription factor to form dimeric or trimeric complexes to enhance the specificity and affinity of DNA binding (Chang et al., 1997). There are three mammalian MEIS transcription factors, MEIS 1, MEIS2, and MEIS3, and they are characterized by a three amino acid residue loop insertion between helix 1 and helix 2 of the homeodomain, which is an important feature for protein–protein interactions (Bürglin, 1997).

The expression of *Meis2* in many embryonic tissues has been described in mice, including the forebrain, midbrain, hindbrain, spinal cord, and heart (Machon et al., 2015). In the developing mouse forebrain, it is expressed in both the dorsal and ventral telencephalon—dorsally it is mainly expressed in the VZ, while ventral expression predominates in the SVZ of the LGE and in the striatum, excluding the MGE and globus pallidus (Figure 2) (Su et al., 2022). It has been shown that MEIS2 function is important for proliferation and neuron differentiation in the striatum as well as in the olfactory bulb (Toresson et al., 2000; Agoston et al., 2012). In addition, zebrafish studies have demonstrated a role in the development of the mesencephalon, craniofacial skeleton, and heart (Glickman and Yelon, 2002).

In humans, *MEIS2* is expressed in the proliferative zones of the fetal forebrain as well as in the adult brain (Larsen et al., 2010). Interestingly, microcephaly has been observed (not fully penetrant) in patients with chromosome 15q14 microdeletions, which encompasses *MEIS2* and is a well-known chromosomal cause of palatal defects co-occurring with congenital heart defects and intellectual disability. In a recent study, 8 of 17 patients (47%) with a 15q14 deletion presented with microcephaly compared to 2 of 11 patients (18%) with *de novo* MEIS2 variants (Verheije et al., 2019), suggesting *MEIS2* as a responsible gene for microcephaly. Given that microcephaly is more prevalent in 15q14 deletion than in *de novo* variants or intragenic deletions in *MEIS2* (Verheije et al., 2019), it is possible that unidentified variants in a nearby region have a synergistic influence on the phenotype when combined with *MEIS2* loss or variants.

Due to the embryonic lethality of mice with zygotic inactivation of the *Meis2* allele, it is difficult to assess the brain size defect after birth, although the embryonic brain size (as well as the whole body size) is smaller in null mice when compared to controls (Machon et al., 2015). Conditional depletion of *Meis2* in the forebrain would be a valuable experiment to further elucidate the role of *Meis2* in microcephaly.

#### 2.1.4 *NKX2-1* (NK2 homeobox 1)

*NKX2-1*, also known as thyroid transcription factor 1 (*TTF1*) or thyroid-specific enhancer binding protein (*T/EBP*), belongs to the ANTP class of homeobox genes and is the mammalian homolog of the *Drosophila scarecrow* (*scro*) (Guazzi et al., 1990; Mizuno et al., 1991). The protein contains a homeodomain and an NK2 box domain of 18 amino acid sequences at the C-terminus of the NK2-type proteins, which is an important region for the regulation of transcriptional activity (Uhler et al., 2007). NKX2-1 was first identified as a nuclear protein that binds to the thyroglobulin gene promoter (Civitareale et al., 1989; Carré et al., 2009) and is known to play a role in telencephalon and diencephalon development as well as in thyroid, lung, and pituitary development (Guazzi et al., 1990; Mizuno et al., 1991; Kimura et al., 1996; Sussel et al., 1999).

In the embryonic mouse forebrain, *Nkx2.1* is expressed in the progenitor and post-mitotic cells of the MGE and preoptic area (Figure 2). NKX2-1-positive progenitors give rise to GABAergic neurons that migrate tangentially from the MGE to the neocortex, as well as cholinergic neurons located in the striatum (Anderson et al., 2001; Magno et al., 2017). *Nkx2.1* homozygous mutant mice die at birth with lung and thyroid defects, along with complex malformations of the ventral telencephalon structures, including the basal ganglia, hypothalamus, and pituitary (Kimura et al., 1996; Takuma et al., 1998; Sussel et al., 1999). In these mutant mice, the MGE is respecified into LGE (thus devoid of the mainly MGE-derived globus pallidus, but with the expansion of the LGE-derived striatum) and they have a reduced number of GABAergic neurons in the neocortex and cholinergic neurons in the striatum (Sussel et al., 1999). Studies with conditional knockout mice further demonstrated a role for *Nkx2.1* in interneuron specification and migration (Butt et al., 2008; Nóbrega-Pereira et al., 2008).

In humans, the *NKX2-1* gene is localized on chromosome 14q13. During early development, it is expressed in the prosencephalon (which gives rise to the telencephalon and diencephalon) and thyroid bud, and later in the lung epithelium (Carré et al., 2009). *NKX2-1* mutations have been associated with brain–thyroid–lung syndrome, characterized by benign hereditary chorea (movement disorder), congenital hypothyroidism, and infant respiratory distress symptoms (Willemsen et al., 2005). Chromosome 14q13 deletions (that include *NKX2-1*) as well as nonsense mutations in *NKX2-1* have also been linked to microcephaly that occasionally co-occurs with brain–thyroid–lung syndrome (Carré et al., 2009). For example, three of the six families with 14q13 deletion (0.9–17.9Mb) presented with microcephaly, as well as one of the two cases with a nonsense mutation in *NKX2-1* (c.338G>A, p.Trp113*) (Carré et al., 2009). In another report on two siblings with hypothyroidism and respiratory failure due to the 14q12-13.3 deletion, both were reported to have postnatal microcephaly (Iwatani et al., 2000), further implicating the potential role of *NKX2-1* in microcephaly accompanying brain–thyroid–lung syndrome. Microcephaly manifested in individuals with *NKX2-1* variants suggests two possibilities: either a direct contribution of the loss of NKX2-1 function in the brain or an indirect result from its loss in other places such as the thyroid, hypothalamus, and pituitary, manifesting as an overall systematic growth defect.

#### 2.1.5 *OTX1* (Orthodenticle homeobox 1) and *OTX2*

**Figure 3:**
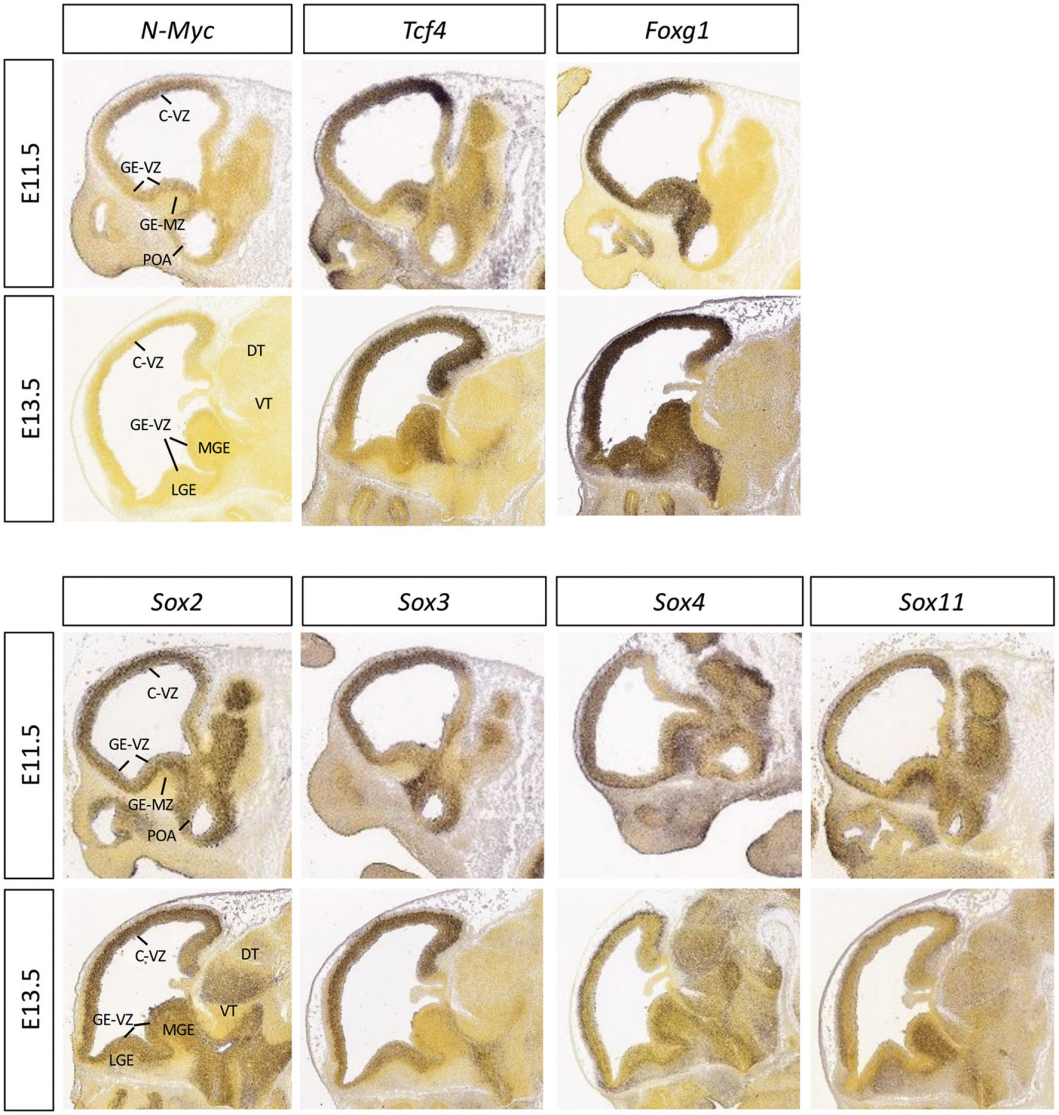
Expression patterns of the selected transcription factors in embryonic mouse brain. The mid-sagittal sections of E11.5 or E13.5 embryonic brains showing RNA *in situ* hybridization results of *N-Myc, Tcf4, Foxg1, Sox2, Sox3, Sox4*, and *Sox11*, adapted from Allen Brain Atlas (https://developingmouse.brain-map.org).

*OTX1* and *OTX2* belong to the PRD class of homeobox genes and are the human orthologs of the *Drosophila orthodenticle* (*otd*) gene, which regulates anterior patterning as well as brain and eye development (Leung et al., 2022). Studies in mice have shown that *Otx2* is essential for the early specification of the rostral neuroectoderm, *Otx1* for corticogenesis, and both for sensory organ development (Simeone, 1998). During early mouse embryogenesis (gastrulation), *Otx2* is expressed in the anterior visceral endoderm and prechordal mesendoderm, which emit signals for early specification and patterning of the neural plate, as well as in the epiblast and anterior neuroectoderm, which respond to these instructing signals (Simeone, 1998; Acampora et al., 1999). *Otx2*-/- null mutant mice are early embryonic lethal and display major abnormalities in their body plan and the absence of the rostral neuroectoderm that normally gives rise to forebrain, midbrain, and rostral hindbrain (Acampora et al., 1995; Matsuo et al., 1995; Ang et al., 1996; Simeone, 1998). On the other hand, *Otx1* expression is first detected at the 1–3 somite stage in the forebrain and midbrain neuroepithelium (Simeone, 1998; Acampora et al., 1999). Its expression in the dorsal telencephalon is restricted to the VZ (where the proliferative, self-renewing, and multipotent neuroepithelial precursors reside) in the earlier stage (Figure 3) but at the end of gestation, it becomes prominent in the cortical plate consisting of layer 5 and 6 neurons, while the VZ expression becomes weaker. *Otx1-/-* null mutant mice mostly die at birth, although 30% survive to weaning age and even into adulthood when on specific genetic backgrounds (Acampora et al., 1996; Suda et al., 1996). The adult null mice that survive have severe morphological anomalies in the eyes, inner ears, pituitary gland, and brain. The weight and size of the brain are reduced; the dorsal telencephalic cortex shows a significant reduction in thickness and cell number, particularly in the temporal and perirhinal cortex, and the cortical lamination is disorganized. These mice also develop spontaneous seizures (Acampora et al., 1996).

These phenotypes in murine models are consistent with those in human patients carrying *OTX1* or *OTX2* variants. In humans, *OTX1* is localized on chromosome 2p15 and *OTX2* on 14q22.3. Mutations in *OTX1*, including chromosomal deletions encompassing *OTX1*, cause developmental delay, short stature, autistic behavior, dysmorphic features, and microcephaly (Liang et al., 2009). For example, one female with the 2p15-16.1 deletion was reported to have all the above-mentioned features, including microcephaly (Liang et al., 2009). In another study, six out of seven patients with variable chromosomal deletion (*OTX1* being the only gene deleted in every case) showed genitourinary defects, and two of these six had microcephaly (Jorgez et al., 2014). Similarly, mutations in *OTX2* have been described in patients with eye defects, variable congenital hypopituitarism, and one individual with microcephaly (Gregory et al., 2021). Despite the low prevalence of microcephaly in patients with *OTX1* or *OTX2* variants, it is worth focusing on these *OTX* genes as potential causal genes for microcephaly, given the severity of the brain phenotypes observed in mice (Acampora et al., 1995; Matsuo et al., 1995; Ang et al., 1996; Simeone, 1998).

#### 2.1.6 *PAX6* (Paired box 6)

*PAX6* is a member of the PRD class of the homeobox gene family and encodes one of the nine PAX transcription factors identified in mammals (Leung et al., 2022). Its *Drosophila* homolog *eyeless* is essential for segmentation, eye and brain development (Noveen et al., 2000; Callaerts et al., 2001; Clements et al., 2009), and in mammals, PAX6 is well known for its important role in the development of the brain, spinal cord, eye, pancreas, and pituitary (Walther and Gruss, 1991; Gehring, 1996). During mouse embryonic development, *Pax6* is expressed in the forebrain, hindbrain, cerebellum, and spinal cord, as well as in the developing eye, pituitary gland, and nasal epithelium (Walther and Gruss, 1991). In the forebrain, *Pax6* is expressed in the proliferating cells in the VZ of the dorsal telencephalon, as well as in the pallial-subpallial boundary, but it is not present in the ventral telencephalon (Figure 2) (Bishop et al., 2000; Hirata et al., 2002).

*Pax6–/–* null mice die at birth with the malformation in the cerebral cortex, and *Pax6*+*/–* mice have thinner cortices with small/reduced eyes (Hill et al., 1991; Tyas et al., 2003; Haubst et al., 2004; Quinn et al., 2007; Mi et al., 2013). Conditional *Pax6* knockout mice with a specific deletion in cortical cells (with Emx1-Cre) have reduced cortical size (Piñon et al., 2008). Explaining these abnormal cortical phenotypes, PAX6 is thought to control the cell cycle length, the transition from symmetrical division to asymmetrical division, and the onset of expression of neural-specific markers, thus regulating the balance between neural progenitor cell proliferation and differentiation. During early neurogenesis, the length of the cell cycle becomes shorter, and the number of S-phase cells (undergoing proliferation) is increased in *Pax6* mutant mice than in the wild type (Estivill-Torrus et al., 2002). Furthermore, the transition from symmetrical (expanding progenitor pool) to asymmetrical (generating post-mitotic cells) division is more rapid in the mutant. As cortical development progresses (E15.5), the cell cycle length becomes longer in the mutant mice. Therefore, the loss of *Pax6* in mice seems to cause depletion of progenitor cell populations in early cortical development as a result of shortened cell length and a faster transition to asymmetrical divisions. However, it should be noted that the cell cycle length change was not detected in another *Pax6* mutant mouse study, although this study found that cell cycle re-entry was reduced (cell cycle exit increased) and the proportion of differentiating neurons was increased (Quinn et al., 2007).

In humans, *PAX6* is located on chromosome 11p13, and its mutations can result in neurological disorders including intellectual disability, autism, impaired audition, and eye defects, as mostly seen in individuals with heterozygous mutations (Malandrini et al., 2001; Davis et al., 2008). Cases with mutations in both alleles of PAX6 (compound heterozygosity) showed severe brain malformations with obvious microcephaly (Glaser et al., 1994; Schmidt-Sidor et al., 2009; Solomon et al., 2009). In these patients, increased germinal proliferation has been described, which is consistent with *Pax6* mutant mouse phenotype that shows increased proliferation in early corticogenesis, which causes depletion of the progenitor pools (Piñon et al., 2008), and other cortical malformations such as polymicrogyria, heterotopia, agenesis of the corpus callosum, holoprosencephaly, etc., as well as defects in eye development (e.g., microphthalmia and aniridia) (Schmidt-Sidor et al., 2009), In addition, 11p13 chromosomal deletions, as well as duplications, encompassing *PAX6* also lead to microcephaly, suggesting that the right level of PAX6, not just the presence of PAX6, is important for proper brain development, although the potential contribution by other genes in the affected chromosome cannot be ruled out.

### 2.2 Basic helix-loop-helix genes: *MYCN* and *TCF4*

Basic helix-loop-helix (bHLH) superfamily genes contain two highly conserved and functionally distinct domains: the “basic domain” at the N-terminus and the “HLH domain” at the C-terminus (Jones, 2004; Murre, 2019). The basic domain binds to DNA at a six-nucleotide consensus sequence (CANNTG) called an E-box; different families of bHLH proteins recognize different E-box consensus sequences (Murre et al., 1989). The HLH domain facilitates protein–protein interactions, forming homo- and hetero-dimeric complexes; many different combinations of dimeric structures are possible with different binding affinities (Fairman et al., 1993). These features of bHLH transcription factors make it possible for them to regulate diverse developmental functions through transcriptional regulation (Murre et al., 1989; Fairman et al., 1993; Jones, 2004), including lineage specification, commitment, self-renewal, proliferation, differentiation, and so on (Murre, 2019). bHLH transcription factors can be grouped into several classes based on expression patterns, DNA-binding specificities, and dimerization selectivity (Murre, 2019). Among them, two members, *MYCN* and *TCF4*, will be highlighted here for their association with microcephaly.

#### 2.2.1 *MYCN* (myelocytomatosis oncogene neuroblastoma derived)

*MYCN* is a member of the *MYC* family of proto-oncogenes and encodes one of the basic helix-loop-helix-zipper (bHLHZ) classes of transcription factors, MYC-N (or N-MYC). The protein forms sequence-specific DNA-binding heterodimers with the bHLHZ protein MAX (Facchini and Penn, 1998; Grandori et al., 2000). The MYC family of proteins is known to be involved in fundamental cellular processes, including proliferation, differentiation, apoptosis, cell growth, and metabolism through regulating target gene transcription. They regulate cell cycle progression-related genes (e.g., CDK4, Cdc25A, cyclin D2, id2, gas1, gadd45, p15^Ink4b^, and p21^Cip1^) (Galaktionov et al., 1996; Lee et al., 1997; Marhin et al., 1997; Bouchard et al., 1999; Perez-Roger et al., 1999; Hermeking et al., 2000; Lasorella et al., 2000; Gartel et al., 2001; Staller et al., 2001), as well as genes controlling cell size and growth, including ribosomal proteins, translation factors, and metabolic enzymes (Rosenwald et al., 1993; Coller et al., 2000; Guo et al., 2000; Boon et al., 2001; Schuhmacher et al., 2001).

*N-Myc* knockout mice are embryonically lethal at mid-gestation (Charron et al., 1992; Stanton et al., 1992; Sawai et al., 1993), and hypomorphic mutations show delayed lethality (Moens et al., 1992, 1993). Conditional knockout mice targeting neuronal progenitor cells display profound microcephaly with small eyes (brain mass reduced 2-folds while body mass reduced 25%) (Knoepfler et al., 2002). In the cKO mouse brains, the VZ thickness and the BrdU incorporation rate were notably decreased, while the proportion of βTubulin III-positive cells (a marker of differentiating and differentiated neurons) increased, suggesting decreased proliferation and increased differentiation (precocious) as the underlying cause of microcephaly (Knoepfler et al., 2002).

In humans, *MYCN* is located on chromosome 2p24.3, and its mutations (loss-of-function) are known to cause type I Feingold syndrome (FS1), which is characterized by variable combinations of microcephaly, limb malformation/digit anomalies, intestinal atresia (blockage/obstruction), and mild to moderate intellectual disability (Courtens et al., 1997; Celli et al., 2003; Bokhoven et al., 2005). Pathogenic variants of *MYCN* are found in ~70% of the patients with FS1; 60% are point mutations, and 10% are chromosomal deletions encompassing the entire *MYCN* locus (Celli et al., 2000; Blaumeiser et al., 2008; Chen et al., 2012; Atik et al., 2016; Tedesco et al., 2021). Microcephaly has been reported in almost 90% of the cases (Bokhoven et al., 2005; Marcelis et al., 2008), which is in line with the small brain phenotype observed in *N-Myc* mutant mice (Knoepfler et al., 2002).

#### 2.2.2 *TCF4* (transcription factor 4)

Another bHLH transcription factor gene, *TCF4*, is highly expressed during brain development (Figure 3) (Jung et al., 2018). This gene (*TCF4*, Gene ID: 6925) is often confused with the transcription factor 7-like 2 gene (Gene ID: 6934, official gene symbol: *TCF7L2*), which is downstream of the WNT pathway and referred to as T-cell factor 4 and often mistakenly abbreviated as *TCF4*. Haploinsufficiency of *TCF4* is known to cause Pitt–Hopkins syndrome, which is characterized by developmental delays with severe intellectual disability, dysmorphic facial features, and episodic hyperventilation and/or breath-holding while awake, among other features. Microcephaly has been reported in up to 60% of Pitt–Hopkins syndrome (Zweier et al., 2008; Goodspeed et al., 2017) and postnatal head growth defect in 26% (Winter et al., 2016). *TCF4* mutations in patients with Pitt–Hopkins syndrome may be deletions, translocations, frameshift, nonsense, or missense mutations (Sepp et al., 2012; Forrest et al., 2014).

Some *Tcf4* mouse models show Pitt–Hopkins syndrome-like phenotypes, including deficits in social interaction and memory as well as abnormal cortical development, neuronal migration, and oligodendrocyte differentiation (Flora et al., 2007; Chen et al., 2016; Kennedy et al., 2016; Thaxton et al., 2018; Li et al., 2019; Wang et al., 2019; Mesman et al., 2020; Wedel et al., 2020). However, mouse lines carrying heterozygous *Tcf4* mutations, which is a clinically more relevant state than homozygous mutations, exhibit mild phenotypes only, without the severe symptoms observed in patients (Papes et al., 2022). A recent study using brain organoids derived from individuals with Pitt–Hopkins syndrome carrying *TCF4* mutations (Papes et al., 2022) demonstrated that neural progenitors bearing these mutations have reduced proliferation and impaired capacity to differentiate into neurons (Papes et al., 2022), providing clear evidence for the role of TCF4 in neural proliferation and differentiation and ultimately microcephaly.

### 2.3 Forkhead box genes: *FOXG1* and *FOXR1*

The forkhead box (FOX) family of transcription factors is named after the ectopic head structures of the *Drosophila* mutants harboring mutations in the *forkhead* (*fkh*) gene. Head involution is blocked in mutant embryos, causing an alteration of the head exoskeleton (called “forkhead”) (Jürgens and Weigel, 1988; Weigel et al., 1989). Since the discovery of *fkh* in *Drosophila* (Hannenhalli and Kaestner, 2009), hundreds of forkhead box genes have been identified from yeasts to humans, and their roles in cell proliferation and cell fate specification have been extensively studied (Carlsson and Mahlapuu, 2002; Lehmann et al., 2003). These transcription factors contain a core forkhead DNA-binding domain (monomeric), which consists of three α-helices connected to a pair of loops resembling butterfly wings or a “winged-helix” (which is why forkhead genes are also called winged helix genes) (Gajiwala and Burley, 2000). The forkhead domain contains ~100 amino acids, sharing no similarity with the previously identified DNA-binding motif (Gajiwala and Burley, 2000). Among several *FOX* genes implicated in human brain development (*FOXG1, FOXC2, FOXL2, FOXP1*, and *FOXP2*) (Lehmann et al., 2003; Golson and Kaestner, 2016), *FOXG1* and *FOXR1* will be highlighted here for their roles in the pathogenesis of microcephaly.

#### 2.3.1 *FOXG1* (Forkhead box G1)

*FOXG1* was originally named brain factor 1 (*BF-1*) when it was identified as the *Hepatocyte Nuclear Factor 3 (HNF-3*) homolog expressed in the developing rat forebrain (Tao and Lai, 1992). Its alterations are known to significantly affect the formation and function of the cerebral cortex (Hannenhalli and Kaestner, 2009; Golson and Kaestner, 2016). In addition to its highly conserved forkhead DNA-binding domain, FOXG1 contains a mammalian-unique N-terminal domain (truncated in non-mammalian vertebrate) and a C-terminal domain responsible for antagonizing the transforming growth factor β (TGF-β) pathway (Hou et al., 2020).

It has been well established that FOXG1 is a master regulator of brain development, controlling cell proliferation (Hanashima et al., 2002), regional patterning (Hanashima et al., 2007; Miyoshi and Fishell, 2012), cell migration (Miyoshi and Fishell, 2012), and circuit assembly (Hanashima et al., 2002; Cargnin et al., 2018). It is strongly expressed in the embryonic forebrain (Figure 3). Its dysfunction leads to multiple congenital brain disorders, including variants of Rett syndrome (FOXG1 syndrome), microcephaly, infantile spasms, autism spectrum disorder, and schizophrenia, and it is associated with various types of cancer (Wang et al., 2018; Hou et al., 2020).

FOXG1's role in brain size control has been revealed through a *Foxg1* knockout mouse model. Constitutive *Foxg1* knockout mice die at birth and present with a severe reduction in the size of the cerebral hemispheres (agenesis of ventral and dorsal telencephalon) (Xuan et al., 1995). Loss of FOXG1 function leads to lengthening of the cell cycle (i.e., reduction in the rate of proliferation) and an increase in cell cycle exit events (i.e., reduction in the fraction of cells that can continue to divide) (Xuan et al., 1995; Hanashima et al., 2002). Premature onset of neuronal differentiation, shown with MAP2 staining, was also detected in *Foxg1* KO mice (Xuan et al., 1995). These results indicate that during normal development, FOXG1 promotes neural stem cell proliferation and suppresses premature neuronal differentiation (Xuan et al., 1995; Martynoga et al., 2005). Studies in cancer models have demonstrated that overexpression of FOXG1 i) inhibited the FOXO/SMAD pathway (which facilitates cortical neuron differentiation), resulting in a reduction in both CDKN1A (cyclin-dependent kinase inhibitor 1A) and cyclin B1 expression, and ii) decreased the proportion of cells in the G2 phase (Wang et al., 2018). Consequently, FOXG1 prevents the cell cycle exit of neural stem cells and promotes stem cell pool expansion. In contrast, FOXG1 knockdown has the opposite effect (Wang et al., 2018).

In humans, haploinsufficiency of *FOXG1* is associated with microcephaly, complete agenesis of the corpus callosum, and cognitive disability (Hou et al., 2020). Patients with FOXG1 mutations also exhibit features of Rett syndrome—a genetic disorder primarily caused by MECP2 mutations—including microcephaly, epilepsy, hyperkinetic movement, impaired sleep patterns, and intellectual disability. However, due to the significant differences in neurological phenotypes of the underlying *FOXG1* mutations (e.g., agenesis of the corpus callosum, blunted gyrification, and reduction in white matter volume in some cases) compared to *MECP2*-mediated Rett syndrome, “FOXG1 syndrome” is now considered a distinct disorder (Kortüm et al., 2011). Microcephaly is one of the three core features of *FOXG1* syndrome, along with agenesis of the corpus callosum and delayed myelination. Patients presenting with these core features have heterozygous variants of *FOXG1* ranging from truncation, frameshift, missense, and nonsense mutations to duplications in the 14q12 *FOXG1* locus (Yeung et al., 2009; Brunetti-Pierri et al., 2011; Seltzer et al., 2014), suggesting the homozygous mutation state is likely lethal, as observed in mice.

#### 2.3.2 *FOXR1* (Forkhead box R1)

*FOXR1*, also known as *FOXN5* or *DLNB13*, is a highly conserved FOX gene containing a forkhead DNA-binding domain. It is expressed in all brain regions during embryonic and postnatal development and also in the reproductive organs based on the human brain transcriptome analysis (Mota et al., 2021). Consistent with the human expression data, mouse *Foxr1* mRNA is also detected in the brain and other tissues (Mota et al., 2021). Although little is known about its function, a study in zebrafish showed that *Foxr1* is an essential maternal effect gene required for proper cell division and survival (Cheung et al., 2018). In agreement with zebrafish findings, *Foxr1* knockout mice showed a severe survival deficit with embryonic lethality in some mice and progressive death in surviving ones (~34% of knockout mice survived to P0 and 23.5% to weaning age). The analysis of newborn mutant mice found cortical thinning with enlarged ventricles (Mota et al., 2021). Together, these results suggest that *Foxr1* is required for survival and normal cortical development.

Recently, a single *de novo* missense variant in *FOXR1* (M280L) in an individual with severe neurological symptoms, including postnatal microcephaly, progressive brain atrophy, and global developmental delay, has been reported (Mota et al., 2021). The phenotypes described in this individual are consistent with those detected in *Foxr1* knockout mice, suggesting that *FOXR1* would be a responsible gene for the observed phenotypes, including microcephaly; however, validation in additional cases is warranted before drawing definitive conclusions.

It is well established that the FOX family of transcription factors induces heat shock protein (HSP, chaperone proteins that prevent protein misfolding) expression, thus providing cells with protective machinery against environmental stressors. FOXR1 appears to play this role as well, given that its most responsive target genes are two members of the HSP70 family (*HSPA1A* and *HSPA6*) and a mitochondrial reductase enzyme, *DHRS2*, each of which plays a role in protective stress response (Mota et al., 2021). The aforementioned M280L variant compromises FOXR1's ability to respond to cellular stressors. Interestingly, some of the upregulated genes by FOXR1 overexpression are involved in ribosome biogenesis (e.g., ribosome biogenesis regulator 1 and *RRS1*), an essential driver in neurodevelopment, whose dysregulation is associated with microcephaly and other neurodevelopmental syndromes (Hetman and Slomnicki, 2019). It is possible that during normal development, FOXR1 plays a role in protecting against proteotoxic stress during ribosome assembly—an energy-demanding process that, if disrupted, can lead to proteotoxic stress in cells (Albert et al., 2019), but further investigations are warranted to unravel the mechanisms underlying FOXR1-associated microcephaly.

### 2.4 *SRY*-related high mobility group box genes: *SOX2, SOX3, SOX4*, and *SOX11*

The *SOX* (sex-determining region Y-related high mobility group box) gene family members encode SOX transcription factors, which belong to the HMG (high mobility group) box superfamily of DNA-binding proteins (Stevanovic et al., 2021). They were first identified based on their high acidic and basic amino acid content and high mobility during polyacrylamide gel electrophoresis (Goodwin et al., 1973). HMG box is a conserved domain in HMG proteins and is responsible for DNA-binding activity. While all HMG family members share a similar HMG box that recognizes DNA structure without apparent sequence specificity, only SOX (and TCF) proteins carry HMG boxes with sequence-specific DNA-binding ability, and they are considered non-canonical HMG proteins (Bustin, 2001).

Based on the structure, expression profiles, and similarity between the proteins they encode, SOX gene family members can be divided into eight groups (A to H), with group B further divided into B1 and B2 (Stevanovic et al., 2021). Within the same group, SOX proteins have an overall high degree of homology, both within and outside the HMG domain, and they have functional redundancy. In contrast, SOX proteins from different groups show poor amino acid sequence homology, especially outside of the HMG domain, and do not show functional redundancy (Wegner, 1999; Bowles et al., 2000). In this study, I will focus on two members of the SOXB1 group (SOX2 and SOX3) and two members of the SOXC group (SOX4 and SOX11) for their associations with microcephaly.

#### 2.4.1 *SOX2* and *SOX3*

*SOX2* and *SOX3* belong to the *SOXB1* group (together with *SOX1*) and were first identified in the screen for homologous genes to the sex-determining gene Sry, which contains an HMG box domain (Gubbay et al., 1990). At the neural induction stage, *Sox2* and *Sox3* are expressed prominently in the neuroectodermal cells, and when neurogenesis begins, it becomes restricted to the VZ, where the proliferative neuroepithelial precursors reside (Figure 3) (Collignon et al., 1996; Guth and Wegner, 2008). At later stages of brain development, their expression is restricted to distinct subsets of mature neurons (e.g., GABAergic neurons in the cortex, striatum, and thalamus) (Cavallaro et al., 2008). Overlapping expression patterns between *Sox1, Sox2*, and *Sox3* appear to suggest a redundant role during CNS development (Uwanogho et al., 1995).

Studies with targeted null mice have demonstrated that *Sox2* functions in the maintenance of the early pluripotent stem cells of the epiblast (Avilion et al., 2003) as well as neural stem cells and their differentiation into neurons in the brain and eye (Pevny and Placzek, 2005). It is also well known for its ability to re-establish pluripotency in terminally differentiated cells by reprogramming them into induced pluripotent stem cells (Takahashi and Yamanaka, 2006). For Sox3, when mouse ES cells targeted with *Sox3* null mutations were injected into blastocysts, the chimeras showed early lethality due to a gastrulation defect (Rizzoti et al., 2004). Although one-third of the *Sox3* conditional knockouts were normal, some exhibited lethality around weaning age with craniofacial defects, reduced size, and decreased fertility (Rizzoti et al., 2004). The analyses of these mice demonstrated that *Sox3* is required for normal pituitary function and the formation of the hypothalamic-pituitary axis. *In ovo* electroporation of the fusion protein between *Sox3* HMG domain and VP16 (transcription activation domain of the viral protein VP16) (HMG-VP16) or EnR (transcription repression domain of the *D. melanogaster* Engrailed protein) (HMG-EnR) into chick embryos revealed that SOX3 is necessary for the formation of neuroectoderm, maintenance of the neural progenitor state, and suppression of neuronal differentiation (Bylund et al., 2003; Schneider et al., 2009). Furthermore, it has been shown that the downregulation of *Sox1, Sox2*, and *Sox3* gene expression by the proneural gene (Neurogenin 2) is essential for neuronal differentiation (Bylund et al., 2003).

In humans, individuals carrying heterozygous loss-of-function mutations in *SOX2* mainly have ocular phenotypes (anophthalmia/microphthalmia), but some patients also present with microcephaly and other variable phenotypes, including hippocampal abnormalities, epilepsy, and motor problems, as well as dysmorphic facial features and genital anomalies (Fantes et al., 2003; Sisodiya et al., 2006; Schneider et al., 2009; Blackburn et al., 2018). The variability in phenotypes points to the complex interactions of SOX2 with other genetic factors, which affect the outcome of SOX2 deficiency in different ways. Similarly, *SOX3* genetic variants have been reported to result in microcephaly with variable penetrance (Jelsig et al., 2018), in addition to hypopituitarism (ranging from isolated growth hormone deficiency to panhypopituitarism), intellectual disability, neural tube defects, and craniofacial abnormalities (Rizzoti et al., 2004; Arya et al., 2020). Notably, the most common mutations identified are duplications or deletions of the whole or part of *SOX3*, with very few examples of point mutations (Jelsig et al., 2018; Li et al., 2022). The microcephaly in some patients with *SOX3* mutations may be the result of growth hormone deficiency due to hypopituitarism as well as neurogenesis defects.

#### 2.4.2 *SOX4* and *SOX11*

*SOX4* and *SOX11* belong to the *SOXC* transcription factor subfamily of genes that are necessary for the survival of neural precursor cells (Bhattaram et al., 2010) and the establishment of their neuronal properties (Bhattaram et al., 2010). In contrast to SOXB1s (SOX1-3), which are expressed in neural precursor cells, SOXC transcripts and proteins (SOX4, SOX11, and SOX12) are mostly expressed in neural cells committed to neuronal differentiation as well as uncommitted precursors (Figure 3) (Hargrave et al., 1997; Kuhlbrodt et al., 1998; Dy et al., 2008). The loss of either *Sox4* or *Sox11* leads to embryonic or postnatal lethality and many other developmental disturbances, although the nervous system defects are not as severe, likely due to their functional redundancy (Schilham et al., 1996; Cheung et al., 2000; Sock et al., 2004; Hoser et al., 2008). Sox11 conditional knockout mice have reduced body size and small brains with reduced cortical thickness noted as early as E11.5 and persisting to birth (Sock et al., 2004; Wang et al., 2013). Forced expression of *SOX11* results in premature induction of neuronal markers, while its deficiency induces apoptosis in the developing nervous system (Bergsland et al., 2006; Bhattaram et al., 2010; Thein et al., 2010). Mice engineered to be conditionally mutant for both *Sox4* and *Sox11* die at birth with microcephaly and an ear phenotype (Gnedeva and Hudspeth, 2015).

In humans, *SOX4* and *SOX11* heterozygous variants have been described in patients with neurodevelopmental syndromes, mild dysmorphisms, and other variable anomalies (Tsurusaki et al., 2014; Hempel et al., 2016; Zawerton et al., 2019; Wakim et al., 2020). The disease phenotypes caused by these two genes are similar, which is consistent with findings in animal models, demonstrating *SOX4* and *SOX11* are co-expressed in various progenitor cell types and have additive or redundant roles in many developing organs including the brain, skeleton, heart, and eye (Angelozzi et al., 2022). *SOX4*- and *SOX11*-related syndromes often share some common features with Coffin–Siris syndrome, which is characterized by abnormal head size (microcephaly or macrocephaly) with characteristic facial features, digits, and eye abnormalities (Angelozzi et al., 2022). Coffin–Siris syndrome is also called “BAFopathy” because its causal genes encode chromatin modeling BAF complex components (e.g., ARID1B and SMARCB1) (Vasko et al., 2021). *SOX4* and *SOX11* are BAF-complex targets (Feng et al., 2013), which accounts for why *SOX4*- and *SOX11*-related syndromes are similar to Coffin–Siris syndrome (Angelozzi et al., 2022). As in other *SOX* gene-associated brain size defects, the microcephaly phenotype observed in individuals with *SOX4* or *SOX11* variants has low penetrance, suggesting a complex interplay between *SOX* genes and other unidentified genes that variably affect the consequences of the *SOX* gene deficiency.

### 2.5 Zinc finger genes: *ZNF238* and *ZNF 335*

Zinc finger proteins (ZNFs) contain a zinc finger domain that can interact with DNA, RNA, PAR (poly-ADP-ribose), and other proteins and are involved in a wide range of cellular processes such as transcriptional regulation, ubiquitin-mediated protein degradation, signal transduction, actin targeting, DNA repair, and cell migration, among others (Cassandri et al., 2017). In this review, only those with transcriptional activity will be focused on, especially the ones associated with microcephaly.

Zinc finger transcription factors constitute the largest family of transcription factors in the human genome. The zinc finger structure is maintained by the zinc ion, which coordinates cysteine (C) and histidine (H) in different combinations (e.g., classical zinc finger has C2H2; non-classical zinc fingers have C2-HC, C2-CH, and C2-C2) (Cassandri et al., 2017). Furthermore, zinc finger motifs can be classified into several different types based on their main-chain conformation and secondary structure around their zinc-binding sites (Krishna et al., 2003; Jen and Wang, 2016). In addition to these zinc motifs, zinc finger transcription factors contain several domains that play different roles in cellular processes, including BTB (Broad-Complex, Tram tracks, and Bric-a-brac), also known as the POZ (poxvirus zinc finger) domain, KRAB (Kruppel-Associated Box) domain, SET domain and SCAN (SRE-ZBP, CTfin51, AW-1, and Number 18 cDNA) domain (Jen and Wang, 2016). Due to the diversity of zinc finger motifs and these additional domains, zinc finger transcription factors can play dynamic roles in gene regulation under various cellular environments and extracellular stimuli. In this study, two zinc finger proteins, ZNF238 and ZNF338, will be highlighted.

#### 2.5.1 *ZNF238* (Zinc finger protein, also known as RP58 or ZBTB18)

*ZNF238*, also known as *RP58* encodes a highly conserved (95% homology in the amino acid sequences between humans and mice) transcription factor containing four zinc finger domains (responsible for DNA binding) and a BTB/POZ domain (multifaceted protein–protein interaction motif) (Aoki et al., 1998; Tatard et al., 2010). Deletion of the distal arm of human chromosome-1q, termed “1qter deletion,” “1q4 deletion,” or “terminal 1q deletion”, is linked to microcephaly with agenesis of the corpus callosum (Boland et al., 2007; Hill et al., 2007; Bon et al., 2008), and the patients carrying this deletion have severe intellectual disability and short stature with profound growth defects (Khadija et al., 2022). A critical region contains a handful of genes, including ZNF238 (Boland et al., 2007; Hill et al., 2007; Bon et al., 2008; Orellana et al., 2010).

During early mouse cortical development (E12.5), *Znf238* expression is detected in a subset of cells in the VZ as well as in the developing neurons in the preplate. At E16.5, while its expression persists in some cells in the VZ, it is also detected in the cortical plate (CP), intermediate zone (IZ), and subventricular zone (SVZ), but not in the marginal zone (MZ). Double-labeling studies with PAX6 and TBR2 showed that the onset of ZNF238 expression coincides with the transition from PAX6-positive cells (radial glial progenitor cells) to TBR2-positive cells (intermediate progenitor cells), the initial stage of intermediate progenitor cells (Okado et al., 2009). Constitutive *Znf238* knockout mice die at birth and show dysplasia of the neocortex and hippocampus, a reduction in the number of cortical neurons, and abnormal laminar organization (Okado et al., 2009). The increased cell death in post-mitotic zones and the expansion of VZ/SVZ in the knockout mice support that ZNF238 is required for the survival and maturation of neurons (excitatory neurons in particular) in the cortex (Okado et al., 2009). Conditional knockout mice with CNS-specific loss exhibit profound postnatal microencephaly, agenesis of the corpus callosum, and cerebellar hypoplasia, which resembles the human phenotype of 1qter deletion syndrome (Xiang et al., 2012), supporting *ZNF238* as a critical, responsible gene for 1qter deletion syndrome whose main features include microcephaly. However, other genes in the deleted region may also contribute to the microcephaly phenotype in 1qter deletion syndrome (Boland et al., 2007; Hill et al., 2007; Bon et al., 2008; Orellana et al., 2010).

#### 2.5.2 *ZNF335* (Zinc finger protein 335, also known as NIF1)

*ZNF335* (*NIF1*) encodes a nuclear zinc finger protein known as a coregulator of nuclear hormone signaling and part of the H3K4 methyltransferase complex (Garapaty et al., 2009). Autosomal recessive primary microcephaly 10 (MCPH10) is caused by a homozygous or compound heterozygous mutation in this gene on chromosome 20q13 (Jayaraman et al., 2018; Lim and Golden, 2020). It is essential for methylation and expression of brain-specific genes, and one of the critical downstream genes of ZNF335 is the master progenitor regulator REST/NRSF [repressor element 1 (RE1)-silencing transcription factor (REST)/neuron-restrictive silencer factor (NRSF)] (Yang et al., 2012). *ZNF335* mutations were first identified in a large consanguineous Arab Israeli family where seven individuals presented with one of the most severe microcephalies reported (9 SD below mean) and death in all except one (Yang et al., 2012). Extremely small brain size with severely simplified gyrification was revealed by MRI, and histopathological analyses demonstrated a thinned cerebral cortex and neuronal disorganization, with only ~20% of the cortex showing the normal six cortical layers (Yang et al., 2012).

In agreement with findings in humans, *Znf335* null mutant mice also show embryonic lethality as early as E7.5, and conditional KO leads to severely reduced cortical size and abnormal cortical layers (Yang et al., 2012). Knockdown of ZNF335 disrupts progenitor cell proliferation (premature cell cycle exit causing precocious depletion of the progenitor pool), cell fate determination, and neuronal differentiation, indicating that ZNF335 is essential for these processes (Yang et al., 2012).

## 3 Discussion/conclusion

In this review, selected transcription factors that are associated with microcephaly have been discussed and summarized. These transcription factors contain unique amino acid domains or motifs responsible for DNA binding (recognizing specific sequences or structures) and protein–protein interactions. Genetic variants of these transcription factors cause a spectrum of neurodevelopmental conditions ranging from mild learning disabilities without obvious structural abnormalities of the brain to severe brain malformations (e.g., microcephaly, lissencephaly, and polymicrogyria) resulting in lethality during early life.

In understanding the pathophysiologic mechanisms underlying microcephaly, knockout mouse models have been useful. Accumulating data suggest that transcription factors can control brain growth and size by directly regulating the expression of the target genes that act on cycle progression, cell cycle exit, neurogenesis, or cell survival (e.g., Galaktionov et al., 1996; Colasante et al., 2015). When target gene expression is compromised due to mutations in transcription factors (e.g., ARX and PAX6), the most common consequences appear to be either an abnormal increase or decrease in neural precursor cell proliferation, and premature cell cycle exit (Estivill-Torrus et al., 2002; Colasante et al., 2015). These defects can lead to the depletion of the self-renewable stem cell populations or a reduction in the number of neurons generated, eventually resulting in microcephaly. In other cases, microcephaly seems to occur as a secondary consequence of overall growth defects (due to growth hormone deficiency, etc.), as observed in hypopituitarism or hypothyroidism caused by mutations in some transcription factors (e.g., NK2.1, OTX2, and SOX3). Furthermore, some transcription factors (e.g., MYCN) regulate the genes important for cell size and growth, such as ribosomal proteins, translation initiation or elongation factors, and metabolic enzymes (Rosenwald et al., 1993; Coller et al., 2000; Guo et al., 2000; Boon et al., 2001; Schuhmacher et al., 2001).

Patients with microcephaly associated with the genetic variants in these transcription factors often exhibit variable penetrance in the severity of the phenotype and in other features co-occurring, which seem to arise from specific types of genetic variants. For instance, mutations causing premature termination (e.g., deletions, nonsense mutations, and frameshift mutations) or missense mutations in critical domains such as homeodomains tend to give rise to a more severe microcephaly with higher prevalence. Alternatively, it is also possible that these variabilities could arise from the involvement of additional genetic interactors that could affect the severity or prevalence of a transcription factor-associated microcephaly (i.e., an unidentified mutation in another gene in addition to the mutation in a given transcription factor).

The knowledge we have built from the studies of individual transcription factors will further our effort to understand brain development in a more systematic way, focusing on the orchestration among all the transcription factors in transcriptional networks. Given that most transcription factors (~75%) work as heterodimers (Walhout, 2006), it will be important to map out interactions between transcription factors involved in microcephaly and to delineate the consequences of their interactions (e.g., changes in DNA-binding preference, changes in DNA-binding affinity, and changes in activity mode between transcriptional activation and repression). Furthermore, systematic analysis and comparison of their target genes using transcriptome analysis (by RNA-seq) and chromatin immunoprecipitation followed by sequencing (ChIP-seq) will provide significant tools to elucidate the role of the transcriptional network in the pathogenesis of microcephaly.

## Author contributions

YL: Writing – original draft, Writing – review & editing.
